# Supplementary material for: Exploration is associated with socioeconomic disparities in learning and academic achievement in adolescence
Source: Nat Commun. 2025 Jul 9;16:6342. doi: 10.1038/s41467-025-61746-6 (PMC12241540; doi:10.1038/s41467-025-61746-6)
Supplement: Supplementary file 1 — Supplementary Information [file 41467_2025_61746_MOESM1_ESM.pdf]

Supplementary Information

Exploration is Associated with Socioeconomic Disparities in Learning and Academic Achievement in Adolescence

Alexandra L. Decker, Julia Leonard[+], Rachel Romeo[+], Joseph Itiat, Nicholas A. Hubbard, Clemens C.C. Bauer, Hannah Grotzinger, Melissa A. Giebler, Yesi Camacho Torres, Andrea Imhof, and John D. E. Gabrieli

[+]Denotes equal contribution

Corresponding Author:
Alexandra L. Decker
Email: adecker@mit.edu

Supplementary Note 1

Adolescents Exhibited Learning on Long and Short Balloon Trials

In the primary manuscript, we observed that adolescents earned more points with experience on long and short balloon trials, suggesting incremental learning. In supplementary analyses, we further probed whether adolescents demonstrated learning by examining the relationship between trial number, pumps and explosions.

We observed a positive association between trial number and the number of pumps on long ballon trials (z = 8.49, p < 0.001, b = 0.005, 95% CI [0.004, 0.006]). Thus, adolescents pumped more as they gained experience with the task. In contrast, there was no significant association between trial number and explosions on long balloon trials (z = 0.26, p = 0.798, b = 0.002, 95% CI [-0.012, 0.016]), possibly because the high explosion threshold resulted in fewer explosions, limiting power to detect longitudinal decreases in explosion rates. On the short balloon trials, there was no significant association between trial number and pumping (z = -1.45, p = 0.148, b = -0.001, 95% CI [-0.002, 0.000]), though, adolescents experienced fewer explosions across the task (z = -7.09, p < 0.001, b = -0.020, 95% CI [-0.026, -0.014]). On unreliable balloon trials, there was no statistically

significant association between trial number and the number of pumps (*z* = 0.08, *p* = 0.933, *b* = 0.000, 95% CI [-0.001, 0.001]) or explosions (*z* = -0.82, *p* = 0.415, *b* = -0.003, 95% CI [-0.009, 0.003]). These results suggest that adolescents incrementally learned the explosion threshold of the long and short balloon trials.

Supplementary Note 2

Lower SES Correlated with Less Exploration

In the primary manuscript, we operationalized exploration using pumps and explosions on unreliable balloon trials. We made this decision a priori because we anticipated that individuals would show evidence of learning on the long and short balloon trials, which could bias the individual difference metric of exploration. For thoroughness and to increase the credibility of the findings, we re-fit models relating SES to exploration, but substituted the primary metric of exploration with pumping and explosions on all trial types. We observed the same pattern of results as those reported in the primary paper: Lower SES correlated with less pumping (*z* = 4.31, *p* < 0.001, *b* = 0.099, 95% CI [0.053, 0.144]) and fewer explosions (*z* = 2.83, *p* = 0.005, *b* = 0.18, 95% CI [0.06, 0.30]).

As an exploratory analysis, we also re-fit models to test whether SES was related to pumping and explosions when restricted to long and short balloon trials. On long balloon trials, lower SES was associated with less pumping (*z* = 3.87, *p* < 0.001, *b* = 0.141, 95% CI [0.070, 0.213]), though not fewer explosions (*z* = 1.21, *p* = 0.227, *b* = 0.19, 95% CI [-0.11, 0.49]). Similarly, on short balloon trials, lower SES was linked to less pumping (*z* = 4.04, *p* < 0.001, *b* = 0.046, 95% CI [0.023, 0.068]), though, not fewer explosions (*z* = 1.82, *p* = 0.068, *b* = 0.17, 95% CI [-0.01, 0.35]). On the whole, these findings show that lower SES adolescents secured points earlier and sampled less to the balloon's explosion limits.

Supplementary Note 3

Exploration mediates the relationship between lower SES and reduced task performance in the BELT

We performed secondary analyses, and robustness checks to confirm that exploration mediated SES-based differences in task performance. In this supplement, we repeated analyses in the primary paper, except we used all trial types to operationalize task performance or exploration. We found all results were consistent with the primary results reported in the paper.

First, we re-fit models to test whether pumping and explosions on unreliable balloon trials (the primary metrics of exploration) mediated SES-based differences in points earned in the last third of the task on all trial types (not just long balloon trials). We found that exploratory pumping (mediation effect [ab] = 0.135, p = .001, 95% CI [0.052, 0.240]) and explosions on unreliable balloon trials (mediation effect [ab] = 0.068, p = 0.004, 95% CI [0.016, 0.140]) mediated SES-based disparities in points earned. While SES was related to points earned in both models (pumping model: total effect [c] = 0.285, p < .001, 95% CI[0.114, 0.450]; explosion model: total effect [c] = 0.285, p < .001, 95% CI[0.115, 0.450]), these effects were reduced after accounting for pumping (direct effect [c’] = 0.149, p = 0.063, 95% CI[-0.009, 0.300]) and explosions (direct effect [c’] = 0.217, p = 0.015,95% CI[0.043, 0.380]).

Second, we re-fit the primary models, but operationalized exploration using all trial types rather than just unreliable balloon trials. Specifically, we asked whether the mean number of pumps and explosions across all trials mediated SES-based differences in points earned on long balloon trials in the last third of the task. We found the same pattern of results reported in the primary paper: individual differences in pumping mediated SES-based differences in points earned (mediation effect [ab] = 0.284, p < .001,95% CI[0.143, 0.430]). That is, while SES was related to points earned (total effect [c] = 0.340, p < 0.001, 95% CI[0.178, 0.510]), this relationship was not statistically significant after accounting for pumping (direct effect [c’] = 0.056, p = 0.27, 95% CI[-0.04, 0.15]). Similarly, individual differences in explosions across all trial types mediated SES-based differences in points earned (mediation effect [ab] = 0.071, p = 0.006, 95% CI[0.016, 0.150]). Indeed, while the relationship between SES and points earned was significant in the mediation model (total effect [c] = 0.341, p < 0.001, 95% CI[.172, 0.500]), this relationship was reduced after accounting for explosions (direct effect [c’] = 0.269, p = 0.002, 95% CI[0.104, 0.430]). These findings show that

exploration mediated SES-based differences in task performance and these results were robust to different operationalizations of exploration and task performance.

Supplementary Note 4.

Exploratory pumping mediates SES-based differences in academic achievement

We repeated the analyses testing whether individual differences in exploration mediated SES-based differences in academic achievement, but operationalized exploration as pumping across all trial types (not just unreliable balloon trials). Consistent with our findings reported in the primary paper, individual differences in pumping mediated SES-based differences in academic skills in the lower-SES subgroup (mediation effect [ab] = 0.197, p = 0.004, 95% CI [0.048, 0.400]). Indeed, while SES was related to academic skills (total effect [c] = 0.466, p = 0.008, 95% CI [0.131, 0.790]), this relationship was not statistically significant after accounting for pumping (direct effect [c’] = 0.269, p = 0.100, 95% CI[-0.053, 0.590]). Furthermore, consistent with results in the main paper, individual differences in pumping mediated SES-based differences in grades (mediation effect [ab] = 0.062, p = 0.039, 95% CI[0.003, 0.140]). While SES was related to grades (total effect [c] = 0.290, p = 0.001, 95% CI[0.111, 0.460]), this relationship was reduced significantly after accounting for pumping (direct effect [c] = 0.228, p = 0.019, 95% CI[0.040, 0.41]).

Supplementary Note 5

Loss aversion was not a statistically significant mediator of the relationship between grades and academic skills

We fit 2 exploratory mediation models to test whether loss aversion mediated SES-based differences in grades and academic skills. These analyses revealed that there was no statistically significant evidence that loss aversion mediated SES-based disparities in academic skills in the lower-SES subgroup (mediation effect [ab] = 0.0673, p = 0.376, 95% CI [-0838, 0.24]). That is, higher SES was significantly related to better academic skills (total effect [c] = 0.467, p = 0.007, 95% CI[1.31, 0.800]), and there was a statistically significant reduction in the strength of this relationship after accounting for loss aversion (direct effect [c'] = 0.399, p = 0.033, 95% CI[0.033, 0.75]). Similarly, there

was no statistically significant evidence that loss aversion mediated SES-based differences in grades (mediation effect [ab] = 0.020, p = 0.494, 95% CI [-0.042, 0.090]). Higher SES was significantly related to better grades (total effect [c] = 0.294, p < .001, 95% CI [0.119, 0.480]), and this relationship was not statistically significantly reduced after accounting for loss aversion (direct effect [c'] = 0.270, p = 0.006, 95% CI[0.079, 0.450]).

Supplementary Figures

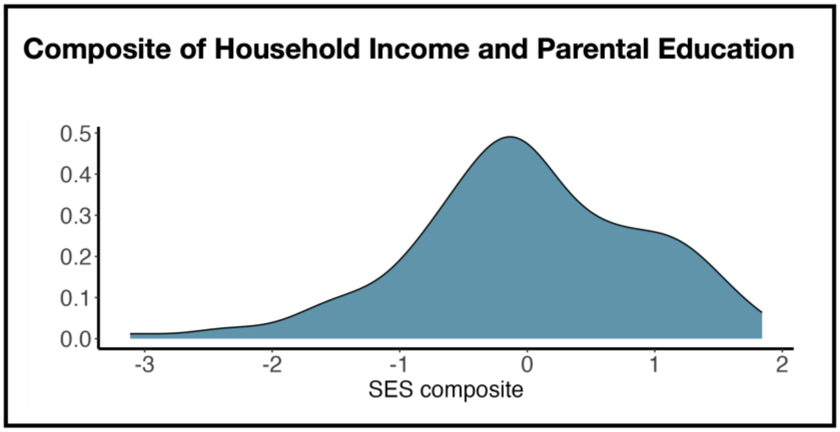

Supplementary Figure 1. Composition SES Score. The Composite SES score, reflecting the mean of the z-score for parental education and log income (n=124). Y-axis reflects proportions.

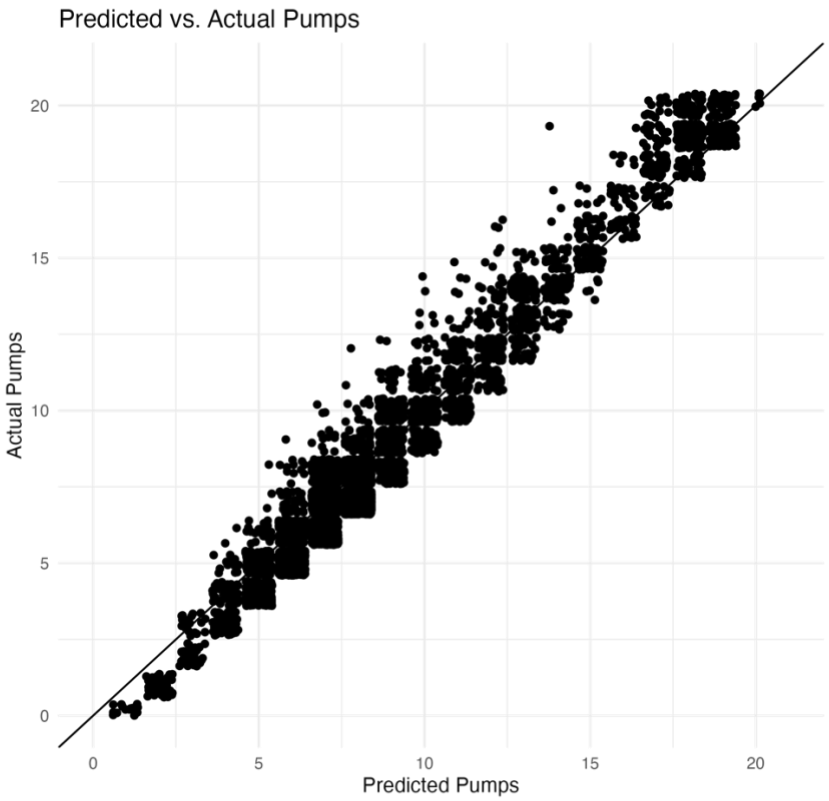

Supplementary Figure 2. Model-Predicted and Actual Behavior. The relationship between the model-predicted number of pumps and the actual number of pumps across participants (n=124). The line reflects the linear regression line of best fit. Statistical Test: Pearson's correlation; r = 0.98, p < 0.001. Each data point is a trial.

Supplementary Tables

Supplementary Table 1. Data on Ethnicity

SES	No. of Subjects	White	Black	Asian	Hispanic	Native American	Pacific Islander	Other
Overall Sample	122	73.77	14.75	7.37	13.93	0.81	0	5.73
Higher SES	58	81.03	6.89	10.34	6.89	1.72	0	5.17
Lower SES	64[&]	67.18	21.87	4.68	20.31	0	0	6.25

Values in each cell reflect a percentage of the sample. Note that some participants reported more than one ethnicity and therefore percentages in each row exceed 100. Subjects were divided into a higher and lower-SES subgroup using a mean split on the SES data. [&]Two participants in the lower-SES subgroup did not report ethnicity data. Thus, the number of participants that reported ethnicity data was 122 (out of 124 included in the sample). Note that these numbers are comparable to the population of the United States (https://www.census.gov/quickfacts/), which is comprised of 75.3% White, 13.7% Black, 6.4% Asian, 19.5% Hispanic or Latino, 1.3% Native American, and 0.3% Pacific Islander.

Supplementary Table 2. Reading or Language Difficulties or Delays

SES	No. of Subjects*	Dyslexia or another reading difficulty or delay	Language difficulty or delay
Overall Sample	124	3*	2
Higher SES	58	0	0
Lower SES	66	3*	2

Values in each cell reflect the number of participants in the sample that reported either a reading or a language difficulty or delay. *Two participants who reported a reading difficulty or delay also reported a language difficulty or delay. The total number of participants with either a reading or a language difficulty or delay was 3 (of 124). Subjects were divided into a higher and lower-SES subgroup using a mean split on the SES data.
