# Supplementary material for: Exploration is associated with socioeconomic disparities in learning and academic achievement in adolescence
Source: Nat Commun. 2025 Jul 9;16:6342. doi: 10.1038/s41467-025-61746-6 (PMC12241540; doi:10.1038/s41467-025-61746-6)
Supplement: Supplementary file 2 — Reporting Summary [file 41467_2025_61746_MOESM2_ESM.pdf]

nature portfolio

Corresponding author(s): Alexandra Decker

Last updated by author(s): 2025/05/25

Reporting Summary

Nature Portfolio wishes to improve the reproducibility of the work that we publish. This form provides structure for consistency and transparency in reporting. For further information on Nature Portfolio policies, see our Editorial Policies and the Editorial Policy Checklist.

Statistics

For all statistical analyses, confirm that the following items are present in the figure legend, table legend, main text, or Methods section.

n/a	Confirmed	
☐	☒	The exact sample size (*n*) for each experimental group/condition, given as a discrete number and unit of measurement
☐	☒	A statement on whether measurements were taken from distinct samples or whether the same sample was measured repeatedly
☐	☒	The statistical test(s) used AND whether they are one- or two-sided<br>*Only common tests should be described solely by name; describe more complex techniques in the Methods section.*
☐	☒	A description of all covariates tested
☐	☒	A description of any assumptions or corrections, such as tests of normality and adjustment for multiple comparisons
☐	☒	A full description of the statistical parameters including central tendency (e.g. means) or other basic estimates (e.g. regression coefficient) AND variation (e.g. standard deviation) or associated estimates of uncertainty (e.g. confidence intervals)
☐	☒	For null hypothesis testing, the test statistic (e.g. *F*, *t*, *r*) with confidence intervals, effect sizes, degrees of freedom and *P* value noted<br>*Give P values as exact values whenever suitable.*
☐	☒	For Bayesian analysis, information on the choice of priors and Markov chain Monte Carlo settings
☐	☒	For hierarchical and complex designs, identification of the appropriate level for tests and full reporting of outcomes
☐	☒	Estimates of effect sizes (e.g. Cohen's *d*, Pearson's *r*), indicating how they were calculated

Our web collection on statistics for biologists contains articles on many of the points above.

Software and code

Policy information about availability of computer code

Data collection	Data was collected using Psychopy Version 2
Data analysis	Statistical analyses were performed in R (version 4.2.2).

For manuscripts utilizing custom algorithms or software that are central to the research but not yet described in published literature, software must be made available to editors and reviewers. We strongly encourage code deposition in a community repository (e.g. GitHub). See the Nature Portfolio guidelines for submitting code & software for further information.

Data

Policy information about availability of data

All manuscripts must include a data availability statement. This statement should provide the following information, where applicable:

- Accession codes, unique identifiers, or web links for publicly available datasets
- A description of any restrictions on data availability
- For clinical datasets or third party data, please ensure that the statement adheres to our policy

Data for this article is publicly available at the following link: https://osf.io/hwt6z/

Research involving human participants, their data, or biological material

Policy information about studies with human participants or human data. See also policy information about sex, gender (identity/presentation), and sexual orientation and race, ethnicity and racism.

Reporting on sex and gender	We collected data on biological sex (62 male and 62 female) and therefore use the term sex in the manuscript. Sex was determined based on self-report. The findings were derived from both sexes. We have reported sex-disaggregated participant numbers in both the Methods section and separately for the higher- and lower-SES subgroup. Furthermore, we have included data on sex in the source data files on OSF.<br><br>Sex and gender were not considered in the study design or hypotheses. Following the journal's guidance discouraging post-hoc sex-based analyses when underpowered, we did not conduct post-hoc sex-based analyses. Our a priori power calculations determined that 100 participants were needed for detecting medium to large effects reported in the literature (d = .53) with 80% power. Sex-stratified analyses with 62 participants per group provides only 50% power to detect these medium effects, and therefore would be underpowered, increasing the risk of type II errors and misleading conclusions.<br><br>We state the following in the manuscript: "Sex was not considered in the study design and no sex-based analyses were conducted as the study was not powered to detect sex differences and this was not a focus of the research."
Reporting on race, ethnicity, or other socially relevant groupings	Socioeconomic status (SES) served as the primary socially constructed covariate. SES was determined through parent self-reported family income and parental years of education, with household incomes ranging from $2,000 to $1.25 million annually (median: $100,000) and parental education levels varying with maternal education ranging from 7-20 years (median: 16 years) and paternal education ranging from 7-20 years (median: 14 years). SES served as a continuous covariate/variable of interest in all analyses related to primary research questions. As detailed in the paper, we derived an SES composite score used in analyses, that gave equal weight to income and education variables. This calculation was equivalent to the mean of z score of the log of the household income data and the z score of the average family years of education variable.<br><br>In addition, some analyses included SES as a categorical variable broken down by higher and lower SES-subgroups, divided using a mean split on the SES data. Details per group are reported in the paper (n = 58 in the higher SES group [median income: $150,000; range: $65,000-$1.25 million; median parental education: 17 years; range: 14-20 years, n = 28 male, n = 30 female] and 66 in the lower SES group [median income: $70,000; range: $2,000-$200,000; median parental education: 13 years; range: 7-17 years, n = 34 male, n = 32 female]).<br><br>We also collected ethnicity data through parent self-report. Relevant categories were: White, Black, Asian, Hispanic, Native American, Pacific Islander, Other). We have included this information in Supplementary Table 1, broken down by higher- and lower-SES subgroups. This demographic data is provided for transparency and to allow readers to evaluate the representativeness of our sample compared to U.S. population demographics. Ethnicity was not included as a variable in our analyses, as examining ethnic differences was not part of our pre-specified research questions or study design. Following rigorous research practices, we avoid conducting post-hoc analyses based on ethnicity, as these were not planned in our original study design and would lead to underpowered comparisons. The source data containing this demographic information is available for reference, maintaining transparency while adhering to methodologically sound analytical approaches. These variables were not used as proxies for SES or other socially constructed variables, nor were they included in our primary analyses as covariates as they were not part of our pre-specified research questions.
Population characteristics	As detailed above, SES was the primary variable of interest. Ethnicity data were also collected through participant self-report and are presented in Supplementary Table 2 for demographic transparency but were not included as covariates in analyses.<br><br>Three participants with documented reading or language delays were included in the sample, with their cases detailed in Supplementary Table 2. Analyses conducted both with and without these participants yielded consistent results.<br><br>All participants were in the 7th or 8th grades (12-14 years old) and age was not included as a covariate because there was minimal age variance in the data.
Recruitment	Participants were recruited from the greater Boston metropolitan area between 2017-2020 using multiple recruitment strategies: distribution of flyers, social media outreach, and direct engagement with local schools. This recruitment approach helped reach a diverse pool of potential participants. However, it is also possible this approach introduced some self-selection bias, as families who responded to recruitment materials may differ systematically from those who did not respond. Additionally, recruitment through schools and social media platforms may have resulted in a sample that over-represents families with higher levels of engagement in their children's activities and research participation.
Ethics oversight	The MIT Committee on the Use of Human Subjects

Note that full information on the approval of the study protocol must also be provided in the manuscript.

Field-specific reporting

Please select the one below that is the best fit for your research. If you are not sure, read the appropriate sections before making your selection.

☐ Life sciences ☒ Behavioural & social sciences ☐ Ecological, evolutionary & environmental sciences

For a reference copy of the document with all sections, see nature.com/documents/nr-reporting-summary-flat.pdf

Behavioural & social sciences study design

All studies must disclose on these points even when the disclosure is negative.

Study description	The study employed a quantitative cross sectional/experimental design examining the relationship between socioeconomic status (SES), exploratory behavior, and task-based performance and academic achievement in adolescents. The study used the Balloon Emotional Learning Task (BELT) to measure exploration and exploitation behaviors across SES, and how performance was related to academic assessments (grades, academic skills).
Research sample	The sample comprised 124 adolescents aged 12-14 years (mean age: 13.46, 62 female, 62 male) recruited from diverse socioeconomic backgrounds in the greater Boston metropolitan area. All participants were enrolled in 7th or 8th grade with household incomes ranging from $2,000 to $1.25 million annually (median: $100,000) and parental education levels varying considerably, with maternal education ranging from 7-20 years (median: 16 years) and paternal education ranging from 7-20 years (median: 14 years). The sample was specifically selected to examine differences across socioeconomic status and efforts were made to ensure the sample was representative of the area. The median household income for the greater Boston area during the data collection period was $113,300 and the median of our sample was similar at $100,000. The ethnicity data, detailed in Supplementary Table 1, also is representative of the United States population (https://www.census.gov/quickfacts/), which is comprised of 75.3% White, 13.7% Black, 6.4% Asian, 19.5% Hispanic or Latino, 1.3% Native American, and 0.3% Pacific Islander).
Sampling strategy	Participants were recruited through multiple channels including flyers, social media outreach, and direct engagement with local schools between 2017 and 2020. This convenience sampling approach helped reach a diverse pool of potential participants, though it may have introduced self-selection bias as families who responded could differ systematically from those who did not respond.<br><br>We aimed to collect data from at least 100 participants based on moderate to large effect sizes of SES and cognitive performance. An a priori power analysis indicated that 100 participants enabled 80% power to identify medium-sized effects (d of 0.57 or Pearson's r of 0.28) in two-tailed individual difference analyses. Furthermore, a sensitivity analysis revealed that 124 participants allowed for 80% power to identify medium-size effects (d of 0.51 or r of .248) in two tailed individual differences analyses.
Data collection	The Balloon Emotional Learning Task (BELT) was administered via computer using PsychoPy version 2. The program automatically recorded participants' choices and reaction times. An experimenter remained present throughout task completion to ensure protocol adherence.<br><br>A trained experimenter administered the Woodcock-Johnson Test of Achievement Edition IV, which provided standardized measures of academic skills. This assessment evaluated word reading, spelling, and mathematical calculation abilities.<br><br>Parents provided the following information through self-report questionnaires: (1) Student grades (on a categorical scale from mostly As to mostly Ds or below); (2) Demographic details including participant age and ethnicity; (3) Presence of any learning disorders; (4) Household socioeconomic information including income and parental education levels<br>Experimenters were not blind to participants' socioeconomic status.
Timing	2017-2020
Data exclusions	One hundred and twenty-nine children and adolescents were recruited. Of the 129 participants recruited, 125 completed the BELT. However, one of the 125 participants did not report parental education or income data and was therefore excluded from analyses. The final sample included 124 participants. There were 124 in all analyses except for those involving school grades as we were grades for two participants. Analysis involving grades included 122 participants.
Non-participation	Of the 129 participants recruited, 4 did not complete the Balloon Emotional Learning Task (BELT) due to fatigue, as the task was part of a larger study that included multiple behavioral assessments and MRI tasks conducted across separate sessions at MIT. These participants elected to discontinue their participation before completing the BELT portion of the study. 1 participant did not report SES.
Randomization	This study did not involve randomization to experimental groups as it examined natural variations in socioeconomic status and their relationship to exploratory behavior and academic achievement.

Reporting for specific materials, systems and methods

We require information from authors about some types of materials, experimental systems and methods used in many studies. Here, indicate whether each material, system or method listed is relevant to your study. If you are not sure if a list item applies to your research, read the appropriate section before selecting a response.

Materials & experimental systems

n/a	Involved in the study
☒	☐ Antibodies
☒	☐ Eukaryotic cell lines
☒	☐ Palaeontology and archaeology
☒	☐ Animals and other organisms
☒	☐ Clinical data
☒	☐ Dual use research of concern
☒	☐ Plants

Methods

n/a	Involved in the study
☒	☐ ChIP-seq
☒	☐ Flow cytometry
☒	☐ MRI-based neuroimaging

Plants

Seed stocks	NA
Novel plant genotypes	NA
Authentication	NA
